# SELF-MANAGEMENT INTERVENTIONS IN DIALYSIS FACILITIES

**DOI:** 10.1093/geroni/igz038.267

**Published:** 2019-11-08

**Authors:** Tiffany R Washington, Chivon Mingo, Matthew L Smith

**Affiliations:** 1 University of Georgia, Athens, Georgia, United States; 2 Georgia State University, Atlanta, Georgia, United States; 3 Texas A&M University, College Station, Texas, United States

## Abstract

There have been few attempts to implement self-management interventions in dialysis facilities. This paper presentation describes four studies that constitute the formative work necessary to inform implementation. Studies one and two examined the relationship between psychosocial factors and kidney disease self-management, finding depression and cognitive decline to undermine self-management behaviors. Study three examined how dialysis patients define and think about self-management and their interest in participating in a self-management program. Among them, 78% affirmed they would participate in a self-management program if it were offered in their facility. Finally, when examining organizational readiness to implement a self-management intervention in dialysis facilities, staff perceived it to be consistent with facility values, and agreed they were well-positioned to implement the program. Taken together, these findings suggest a self-management intervention would be beneficial and supported, but would require thoughtful implementation considerations. These studies have implications for developmental self-management research with other chronic conditions.

